# A single‐center descriptive account of the use of pectoral nerve I and II nerve blocks for post‐operative pain relief following pediatric sternotomy

**DOI:** 10.1002/pne2.12092

**Published:** 2022-12-07

**Authors:** Zachary Freedman, Jacob AuBuchon, Michael Montana

**Affiliations:** ^1^ Penn State College of Medicine Hershey Pennsylvania USA; ^2^ Department of Anesthesiology Washington University in St. Louis and St. Louis Children's Hospital St. Louis Missouri USA

**Keywords:** Pain, PECS Block, Pediatrics, Sternotomy

## Abstract

Regional anesthesia between the pectoralis major and minor was first described in 2011 as an alternative method to paravertebral blocks or epidurals for post‐operative mastectomies. Since then, the use of pectoral nerve (PECS) blocks for post‐operative pain management following thoracotomy, sternotomy, and other procedures in the anterior thorax has increased. While experience with this block is growing, the current understanding of its use in pediatric patients is limited. We reviewed pediatric cases at a single institution and provide a descriptive account of our use of PECS I and II blocks for post‐operative pain management following operations involving sternotomy in pediatric patients. We performed a retrospective database analysis of the use of PECS I and II blocks following procedures requiring sternotomy from 2018 to 2021 at St. Louis Children's Hospital. Patients 21 years old and younger who received either a PECS I or II block following a sternotomy for a cardiac procedure were included in the analysis. Patient's demographics, pre‐, intra‐, and post‐operative medications, operative time, extubation status, pain evaluations, and hospital course were assessed from the electronic medical record. From 2018 to 2021, 73 ultrasound‐guided PECS blocks were performed for pain relief for pediatric sternotomy. The most commonly performed operations were atrial septal defect closure (*n* = 12), mitral valve repair (*n* = 8), and ventricle septal defect closure (*n* = 8). Out of the 73 patients, 47 received a PECS I block and 26 received a PECS II Block. 70 of the blocks were administered after closure of the sternum while 3 were done before incision. The time to perform blocks took on average of 6 (±4) min. Mean operating room time was 7.5 h. Local anesthetics used for the blocks were as follows: Ropivacaine 0.2% (*n* = 54), Ropivacaine 0.5% (*n* = 18), and Bupivacaine 0.25% (*n* = 1). Twenty‐five out of 73 patients did not experience severe pain, defined as ≥7/10 on a numeric pain scale, at any point in the first 24 h following surgery. We describe the of use PECS I and II nerve block following pediatric sternotomy. Blocks were straight forward to perform, and typically took a short amount of time to administer (6 min), when compared to the total operating room time (7.5 h). While this study did not include a comparative group that did not receive a block, 34 percent of patients did not suffer from severe pain in the first 24 h following surgery. Further prospective studies are needed to assess the effectiveness of PECS blocks for pain relief following sternotomy in pediatric patients when compared to current standard of care. PECS blocks may be beneficial for a range of cardiac surgeries that typically result in severe postoperative pain.

## 1 INTRODUCTION

The use of regional anesthesia for post‐operative pain relief has become a staple in the hospital setting. These short procedures aim to decrease post‐operative pain and the use of medications for pain relief. Regional anesthesia can occur throughout the body toward many nerves for a variety of procedures.

Fascial plane blocks between the pectoral muscles have been around for over 10 years and were originally described in 2011 by Blanco, who blocked the lateral and medial pectoral nerves during the perioperative period for breast surgeries.^1^ Since then, the pectoral nerve (PECS) block nomenclature has evolved to be three different types: PECS I, PECS II, and serratus plane block. The PECS I block injects anesthetic in the interfacial plane between the pectoralis major and minor muscles at the 3rd rib, blocking the lateral and medial pectoralis nerves. The PECS II block, which was also developed by Blanco in 2012, is comprised of two injections: the PECS I injection with the addition of an injection in the interfacial plane between the pectoralis minor and serratus anterior muscles at the location of the 4th rib blocking the long thoracic nerve, thoracodorsal nerve, and intercostal nerves (T2–T6).^2^ Lastly in 2013, Blanco described the serratus plane block around the serratus anterior muscle at the 5th rib blocking the intercostal nerves (T2–T6).^3^ These blocks hope to decrease pain in the post‐operative setting around the chest area for a variety of breast related procedures.

Traditionally, post‐operative pain relief options include epidurals and paravertebral blocks at the levels of the operation, parasternal blocks, pecto‐intercostal fascial blocks, transverse thoracic plane blocks, and erector spinae plane blocks.^4^ The PECS block has been well established for its use in breast surgery for the adult population.^5^, ^6^, ^7^ However, there has been a recent push in the last couple of years to find other uses for these types of blocks, especially in cardiac surgeries.^8^ Despite its established efficacy in adults, the current understanding of its use in the pediatric population is limited.

The authors provide a descriptive study of the use of PECS I and PECS II blocks for pediatric sternotomy surgeries.

## 2 METHODS

This was a descriptive retrospective study that utilized the SlicerDicer tool within the Epic electronic medical record to identify patients who were under 21 years of age that were given a PECS I or II block following sternotomy for a cardiac procedure between January 1, 2018, and May 30, 2021, at St. Louis Children's Hospital. SlicerDicer is a tool within EPIC to find specific patients based on specific inclusion and exclusion criteria that has been validated by previous studies.^9^ The resulting patients were then assessed for demographics, pre‐, intra‐, and post‐operative medications, operative time, extubation status, pain evaluations, and hospital course from the electronic medical record. As a result of variable time measurements using a combination of four different validated pain scales (rFLACC, FLACC, Faces, and Numeric) for each patient, a non‐validated combination score was implemented to record pain levels.^10^, ^11^, ^12^, ^13^ Any post‐operative outcomes and/or complications were recorded within 30 days following completion of the surgery. All patients received normal anesthetic nerve block post‐operative care at St. Louis Children's Hospital consisting of pain service follow‐ups, opioids, and non‐opioid analgesics.

## 3 RESULTS

**FIGURE 1:**
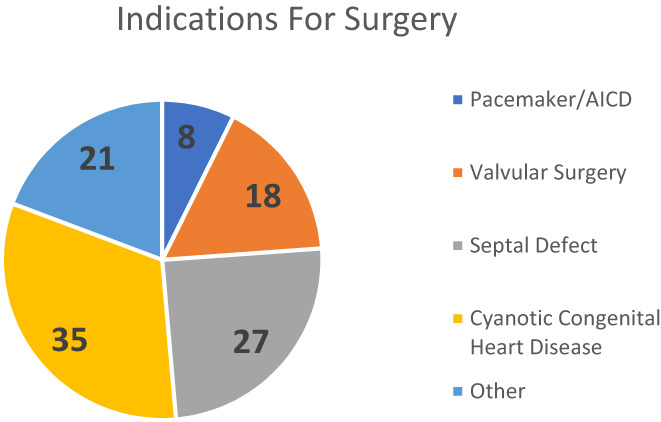
Indications for cardiac surgery among cohort. sum of indications is greater than patients in cohort due to multiple indications per surgery.

In total, 80 patients were obtained from the SlicerDicer search. After assessment of the patients, seven were removed as the blocks were not related to cardiac procedures. Therefore, 73 patients received PECS I or II blocks following sternotomy for cardiac procedures. Out of the 73 patients, 40 were male and 33 were female with an average age of 9 years and 99 days. Fifty‐nine out of 73 patients were classified as American Society of Anesthesiologists (ASA) level 3. The most frequent surgery performed was closure of atrial septal defect at 12 operations and repair of mitral valve and ventricular septal defect at 8. Fifty‐eight patients had a history of congenital heart disease as well. Demographics of the cohort is fully summarized in Table 1. Types of surgery's undergone by patients can be found in Figure 1, and the full list of surgeries can be found in Table S1. Five of the operations performed were due to the need of further procedures in the operating room, and eight of the patients had a previous sternotomy prior to the surgical course being evaluated.

**TABLE 1:** Demographic data of cohort receiving pectoral nerve I or II block

Demographics	
Variables	Cohort (*n* = 73)
Age	9 years 99 days ± 5 years 354 days
Sex	
Male	40
Female	33
Weight (kg)	35.3 ± 23.1
Height (cm) (*n* = 70)	128.3 ± 34.5
BMI (*n* = 70)	18.2 ± 4.4
Race	
White	55
American Indian/Alaska Native	2
Black or African American	12
Asian	4
Ethnicity	
Not Hispanic or Latino	71
Hispanic or Latino	2
ASA classification	
1	1
2	8
3	59
4	5
5	0
History of congenital heart disease	
Yes	58
No	15
History of developmental delay	
Yes	14
No	59

Out of the 73 patients, 66 had same day surgery and seven were inpatients prior to surgery. The average length of stay following surgery in the intensive care unit (ICU) was 2.24 days for same day patients and 1.14 days for inpatients. The average length of stay in the hospital for non‐ICU patients was 4.37 days for same day and 3.71 days for inpatient. The full hospital time course can be found in Table 2. The average length of time spent in the operating room was 7 h and 28 min. Sixty‐five patients received cardiopulmonary bypass during their operations with an average time of 2 h and 3 min. Operating details of the patients can be found in Table 3.

**TABLE 2:** Hospital course for patients receiving pectoral nerve I or II block

Hospital stay	Same day (*n* = 66)	Inpatient (*n* = 7)
Hospital stay pre surgery (Days)		
Average (SD) (Days)	—	8.43 ± 8.85
Maximum	—	28
Minimum	—	3
Days in ICU (Days)		
Average (SD)	2.24 ± 1.63	1.14 ± 0.90
Maximum	10	3
Minimum	0	0
Days on floor		
Average (SD)	4.37 ± 4.57	3.71 ± 1.38
Maximum	28	6
Minimum	0	2
Hospital stay post‐surgery (Days)		
Average (SD) (Days)	—	4.86 ± 1.57
Maximum	—	7
Minimum	—	3
Hospital stay total (Days)		
Average (SD)	6.47 ± 5.41	13.29 ± 9.30
Maximum	38	34
Minimum	2	7

**TABLE 3:** Operating details for patients receiving pectoral nerve I or II block

Operating details	Time in Operating Room (*n* = 73)	Cardiopulmonary Bypass Time (*n* = 65)
Average (SD)	7 h 28 min ± 2 h 13 min	2 h 3 min ± 1 h 3 min
Maximum	12 h 57 min	5 h 12 min
Minimum	3 h 34 min	48 min

**FIGURE 2:**
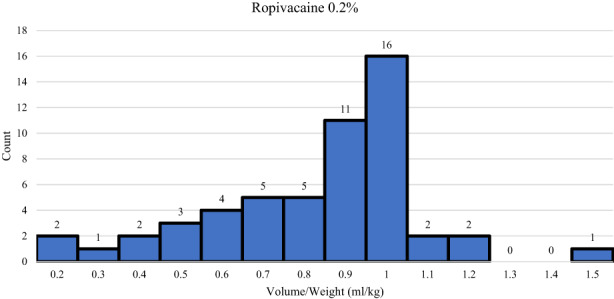
Histogram of patients receiving each volume/weight ratio of ropivacaine 0.2%.

**FIGURE 3:**
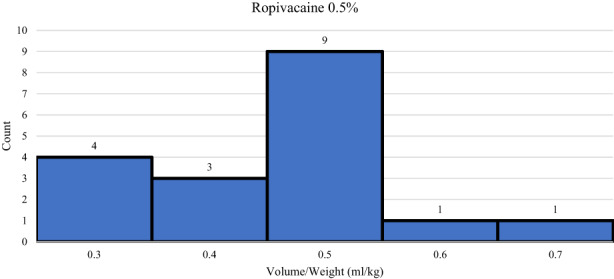
Histogram of patients receiving each volume/weight ratio of ropivacaine 0.5%.

Out of the 73 patients, three received the block pre‐incision and 70 received the block post closure. Those who received the block pre‐incision were due to the expected operating time to short enough that the block would still be effective when they arrived to the post‐operative care unit. After initiation by the team, it took an average of 6.25 min to physically perform the block from ultrasound confirmation to block placement. Within the cohort, 47 patients received a PECS I block and 26 received a PECS II block. Fifty‐four patients received Ropivacaine 0.2% and 18 received Ropivacaine 0.5%. One patient was given Bupivacaine 0.25%. Full details of the PECS Block Placement can be found in Table 4. Average volume/weight dosing of Ropivacaine 0.2% was 0.83 ml/kg, and average volume/weight dosing of Ropivacaine 0.5% was 0.46 ml/kg. The full histogram plots for Ropivacaine 0.2% and Ropivacaine 0.5% can be found in Figures 2 and 3 respectfully. All patients received opioid pain medication and 62 out of 73 patients received anti‐emetic medication during surgery.

**TABLE 4:** Block information for patients receiving pectoral nerve (PECS) I or II block

PECS block	
Variable	Cohort
When was block performed?	
Pre‐incision	3
Post‐closure	70
Time taken to perform block	6.25 min ± 3.37 min
PECS block type	
PECS I	47
PECS II	26
Anesthetic used	
Ropivacaine 0.2%	54
Ropivacaine 0.5%	18
Bupivacaine 0.25%	1

Pain metrics were also recorded from the electronic medical record following surgery. Four pain scales were assessed on patients including rFLACC, FLACC, Faces, and Numeric scoring. Due to no standard protocol implemented on tracking pain levels within the institution, pain records were being done at any time and with whatever scale the nursing staff deemed appropriate at the time. Due to multiple scales being used on the same patient, the numerical value of 1–10 obtained from each scale was used as the pain metric regardless of the scale it came from. For example, a score of 7 on the FLACC scoring scale was equivalent to a score of 7 on the Faces scoring system. Out of the 73 patients described, 25 of them did not experience severe pain defined as ≥7/10 on our combined numeric rating scale at any point in the first 24 h following surgery.

In total, four patients arrived at the ICU still intubated and 69 arrived extubated. Every patient received some type of pain medication in the ICU with an average time of 91 min before administration. The first pain medication given post‐surgery, pain medications given in the first 24 h, and extubation status were recorded in Table 5.

**TABLE 5:** Medication usage in ICU for patients receiving PECS I or II block

Medication Usage in ICU	
Variable	Cohort
Was the patient extubated at the end of the operation?	
Yes	69
No	4
Time in ICU before first pain medication (min)	91 ± 93.2
First pain medication given in ICU	
IV Acetaminophen	9
Rectal Acetaminophen	2
PO Acetaminophen	4
IV Fentanyl	4
IV Hydromorphone	1
IV Methadone	1
IV Methocarbamol	7
IV Morphine	21
Medications given in first 24 h	
IV Acetaminophen	39
Rectal Acetaminophen	13
PO Acetaminophen	39
PO Cyclobenzaprine	2
IV Diazepam	2
IV Fentanyl	3
PO Gabapentin	2
IV Hydromorphone	4
PO Hydrocodone	2
PO Ibuprofen	2
IV Ketorolac	51
Patch Lidocaine	3
IV Methocarbamol	24
IV Methadone	1
PO Morphine	43
IV Morphine	27
IV Nalbuphine	2
PO Oxycodone	20

In the ICU, nine patients reported nausea with five of those patients vomiting. There were no obvious adverse outcomes that we could directly attribute to the administration of the block in the ICU and zero patients were readmitted to the ICU after being admitted to the floor. Only one patient had an emergency room visit related to the surgery within 30 days post discharge.

## 4 DISCUSSION

Our data show that the use of PECS I and II blocks for post‐operative pain relief for pediatric sternotomy operations may be a promising technique in the pediatric population. Congenital heart disease is one of the most common congenital disorders and requires a great deal of post‐operative recovery due to the nature of the disease.^14^ Any type of post‐operative intervention that can add in recovery may be beneficial to this patient population.

Results showed that blocks were straight forward to perform and only took a short time of 6 min compared to the total operating room time of 7.5 h. Despite this study being a descriptive study without a comparison group, these numbers show that this block takes a minimal amount of time to perform and may provide reduced pain in post‐operative recovery for these patients. In addition to the time aspect, no known complications could be directly linked backed to the block which suggests the relatively safe practice of performing this block. Despite these data coming from a single institution, the results can be applicable to other institutions due to the ease of the block performed and the experience of interventional pain teams at academic centers performing these cardiac operations.

An unexpected observation from these data was the increased time spent in the ICU and floor in the same day patients vs the inpatient patients. While outliers within these groups may be a reason for these numbers, the potential recovery time is interesting to note and should be understood by the physician performing the block.

A finding of these data is the need for clinicians and nurses to establish a consistent way to categorize pain. Pain scores in the Electronic Medical Record (EMR) were often recorded at variable time points, using multiple pain scores (rFLACC, FLACC, Faces, Numeric 1–10) within the same patient. There has been much education to our institution to improve this variance. Additionally, some patients had days where no pain score was recorded versus some patients that had a score being recorded every hour. Some patients had gone over 5 h initially in the ICU without a pain score observation. We recognize this may be because the patient is sedated but regardless, a system tracking and utilizing pain scores needs to be established. We also recognize the complexity of pain within the pediatric population and that individuals may not be able to verbally communicate with the care provider. That being said, the appropriate pain scale for varying cognitive levels and ages of patients' needs to be consistent among all providers. As a result of this inability to standardize the pain scores, we were forced to combine all pain scores regardless of measurement as a standard pain score. Despite this, it was found that 25 out of 73 patients did not experience severe pain, defined as ≥7/10 on the multiple pain scale that we had created at any point in the first 24 h following surgery. While the authors recognize the variability in practice of multiple pain scores for each patient, the pain scores recorded are still based on objective measures in the case of rFLACC and FLACC and subjective measures given by the patient in the cases of Faces or Numeric recordings. While this study does provide comparative results to other studies due to the combination of the scoring system utilized, the authors still believe that the measurements still hold validity and accurate pain measurements for the patients involved. A recent report outlining pediatric pain treatment and prevention for hospitalized children stated that pain in patients in children's hospitals is commonly under recognized and under reported.^15^ Additionally sternotomies result in significant post‐operative pain often without sufficient analgesia.^16^ Ultimately, PECS blocks may be a promising technique for post‐operative recovery for pediatric sternotomy operations. Future directions and studies utilizing a control group and a standardized pain scale would be important to compare the benefits of this block.

Literature review shows that despite the established use of PECS for breast surgeries, the understanding of its use in cardiac procedures is in its very early stages. A recent meta‐analysis conducted in 2020 by Jack et al. looking at all literature of PECS block usage found only 9 randomized control trials looking at the use of PECS blocks for cardiac procedures that encompasses only about 600 patients. Of those 9 trials, only one looked the use of PECS II blocks for post sternotomy analgesia. The others were for thoracotomy or video assisted thoracoscopic surgery.^8^ The majority of literature in PECS' block applications is through case series or case reports that were mostly published as conference abstracts.^8^ In addition to this, all the literature focuses on adults rather than in the pediatric population.

The use of an erector spinae plane block has been shown to reduce post‐operative pain following cardiac procedures. However, this block requires the patient to be turned following the operation which can cause an increased turnover time and can add further complications to the patient.^17^ On the contrary, the PECS block provides an appropriate alternative as the patient can be in the same supine position as they were during surgery, which can avoid some of the risks of neuraxial anesthesia such as epidural hematoma.

The PECS block looks promising, but research still needs to be done on the exact analgesic, weight‐based dosing and location of the block. Additionally, provider variability and the exact location on ultrasound of the needle are all confounder variables in the efficacy of the PECS block. An anatomical study on PECS blocks conducted by Versyck et al. described that injection medial to the pectoral branch of the thoracoacromial artery only reaches the medial and lateral pectoral nerves. A lateral approach, which is lateral to the pectoral branch thoracoacromial artery, will also spread to the axilla and reach the intercostobrachial nerve.^18^

A recent double blind randomized controlled study was published that compared bilateral PECS II block vs intravenous analgesics for median sternotomy cardiac surgeries. The findings showed the PECS block group had lower Modified Objective Pain Score (MOPS), 2, 4, and 6 h post‐operatively. The PECS group also had a median utilization of 2.72 mic/kg of fentanyl in the first 24 h compared to 4.17 mic/kg in the control group, a 35% reduction. Despite the efforts within this study, it only involved 40 patients (20 per arm) of children ages 1–5 years old.^19^ Further studies should utilize more patients with a larger age distribution to provide more accurate results.

There were several limitations within this study. The first and most important limitation was the lack of standardization in categorizing pain. Patients had multiple pain scores at varying time points, and it was difficult to gather any sort of trend within the cohort. As a result of the varying pain scales used for each patient, a spaghetti plot that is typical in pain related research was not able to be constructed for the cohort. Further prospective studies should implement 1 pain scale at standard intervals for each patient. An additional limitation is that this work is retrospective, and we are using patient charts and clinician/nursing recordings to interpret results instead of working with and speaking with the patients directly. Lastly, we recognize the subjective nature of pain studies and pain tolerance, especially in pediatric patients who may not be able to effectively communicate with providers.

Future studies are needed to assess the effectiveness of PECS blocks for pain relief following sternotomy in pediatric patients when compared to the current standard of care.

In conclusion, these findings show PECS I and II blocks for pediatric sternotomy as a promising technique for post‐operative pain relief. This study demonstrates the feasibility of the block but unfortunately showed significant variability in pain scores. Future prospective clinical trials should be utilized for the efficacy of this intervention within this population. Lastly, the need to standardize and categorize pain is of great importance to the medical community especially in the development of new techniques for pain management.

## FUNDING INFORMATION

This project was supported by a Medical Student Anesthesia Research Fellowship (MSARF) from the Foundation for Anesthesia Education and Research (FAER) to ZF.

## CONFLICT OF INTEREST

The authors have nothing to disclose.

## Supporting information


Table S1.
