# Supplementary material for: A single‐center descriptive account of the use of pectoral nerve I and II nerve blocks for post‐operative pain relief following pediatric sternotomy
Source: Paediatr Neonatal Pain. 2022 Dec 7;5(1):16–22. doi: 10.1002/pne2.12092 (PMC9997121; doi:10.1002/pne2.12092)
Supplement: Supplementary file 1 — Table S1. [file PNE2-5-16-s001.docx]

**Supplemental:**

Table 1: Indications for Cardiac Surgery Among Cohort. Sum of Indications is Greater Than Patients in Cohort Due to Multiple Indications per Surgery.

| **Procedure** | **Frequency** |
| --- | --- |
| Ablation Radiofrequency | 1 |
| Aortic Valve Leaflet Repair | 1 |
| Bronchoscopy | 2 |
| Closure Atrial Septal Defect | 12 |
| Closure Mitral Valve Cleft | 1 |
| Closure Patent Foramen Ovale | 2 |
| Conduit - Right Ventricle to Pulmonary Artery | 1 |
| Establish Continuity of SVC and Right Pulmonary Artery Reconstruction | 1 |
| Fontan Procedure | 7 |
| Gerbode Defect | 1 |
| Konnors Ventricularplasty (Left Chest) | 1 |
| Left Repair Pulmonary Artery (Left Chest) | 1 |
| Ligation Of Ligamentum Arterioseum/PDA | 1 |
| Ligation Patent Ductus Arteriosus (Left Chest) | 1 |
| Implantable Cardiac Event Monitor Removal | 1 |
| Insertion Epicardial Pacemaker | 1 |
| Insertion Pacemaker | 3 |
| Insertion Pacemaker Dual Chamber Epicardial Pacemaker | 1 |
| Maze Procedure Open Heart (Left Chest) Isolation Left Atrial Appendage | 1 |
| Myectomy - Septal | 1 |
| Patch Repair - Right Ventricular Outflow Tract | 1 |
| Placement Automatic Implantable Cardioverter Defibrillator/Internal Cardioverter Defibrillator | 1 |
| Pulmonary Artery Band Take Down | 1 |
| Reconstruction Pulmonary Artery | 1 |
| Reimplantation of Innominate Artery Via Sternotomy and No Bypass | 1 |
| Removal Mediastinal Mass | 1 |
| Removal Pacemaker Generator/Leads | 1 |
| Resection Right Ventricle Muscle Bundles | 1 |
| Resection Sub Aortic Obstruction | 1 |
| Resection Sub Aortic Stenosis | 1 |
| Resection Subaortic Membrane | 4 |
| Repair Aneurysm Aortic | 1 |
| Repair Aortic Root Fistula | 2 |
| Repair Atrial Septal Defect | 3 |
| Repair Atrioventricular Canal Defect - Partial | 1 |
| Repair Branch Pulmonary Artery Stenosis | 1 |
| Repair Cleft Mitral Valve | 2 |
| Repair Incidental Ventricular Septal Defect | 1 |
| Repair Mitral Valve | 8 |
| Repair of Anomalous Aortic Origin of Coronary Artery - Unroofing | 2 |
| Repair Of Right Ventricular Outflow Tract Muscle Bundle Resection | 1 |
| Repair/Revise Left Ventricular Outflow Tract | 1 |
| Repair Partial Anolmalour Pulmonary Venous Return | 1 |
| Repair Partial Anomalous Pulmonary Venous Return with Sinus Venous Repair | 1 |
| Repair Partial Arteriovenous Canal | 1 |
| Repair Stenosis - Supravalvar Aortic Stenosis | 1 |
| Repair Tetralogy of Fallot | 2 |
| Repair Tricuspid Valve | 3 |
| Repair Ventricular Septal Defect | 8 |
| Replacement Aortic Valve | 1 |
| Replace Pacemaker Battery | 1 |
| Replacement Pacemaker Generator/Leads | 1 |
| Replacement Pulmonary Valve | 2 |
| Right Ventricular Outflow Tract Obstruction Repair | 3 |
| Ross-Konno Procedure | 4 |
| Septal Myomectomy | 2 |
| Shunt - Glenn, Bidirectional Glenn Shunt Takedown | 1 |
| Shunt - Potts | 3 |
